# Oral administration of *Lactobacillus brevis* 23017 combined with ellagic acid attenuates intestinal inflammatory injury caused by *Eimeria* infection by activating the Nrf2/HO-1 antioxidant pathway

**DOI:** 10.1186/s13567-022-01042-z

**Published:** 2022-03-18

**Authors:** Xuelian Yang, Xinghui Pan, Zhipeng Jia, Bingrong Bai, Wenjing Zhi, Hang Chen, Chunli Ma, Dexing Ma

**Affiliations:** 1 College of Veterinary Medicine, Northeast Agricultural University, Harbin, 150030 Heilongjiang China; 2 College of Food Science, Northeast Agricultural University, Harbin, 150030 Heilongjiang China; 3 Heilongjiang Key Laboratory for Experimental Animals and Comparative Medicine, Harbin, 150030 Heilongjiang China

**Keywords:** *Lactobacillus brevis*, ellagic acid, Nrf2/HO-1, ChTLR15/ChNLRP3, inflammatory injury

## Abstract

The aim of this study was to investigate whether oral administration of *Lactobacillus brevis* 23017 (LB) alone and in combination with ellagic acid inhibits ChTLR15/ChNLRP3/ChIL-1β by activating the Nrf2/HO-1 pathway to attenuate intestinal inflammatory injury. Two animal experiments were performed. In Experiment 1, chickens were allocated into 7 groups: PBS, and low, medium and high dosages of live and heat-killed LB, named L/LB(+), M/LB(+) and H/LB(+), and L/LB(−), M/LB(−) and H/LB(−), respectively. In Experiment 2, chickens were divided into 5 groups: PBS, challenge control, and low, medium and high dosages of ellagic acid combined with LB(+), named L/EA + L/LB(+), M/EA + M/LB(+) and H/EA + H/LB(+), respectively. Chickens were gavaged with LB with or without ellagic acid once a day. Then, the mRNA and protein levels of the components of the Nrf2/HO-1 pathway found in the caecal tissues were quantified. On Day 7 post-infection with *E. tenella*, the levels of the components of the ChTLR15/NLRP3/IL-1β pathway in the caeca were again quantified, and the anticoccidial effects were assessed. The results showed that the levels of the genes in the Nrf2/HO-1 pathway in the chickens in the LB(+) groups were higher than those in the LB(−) groups (*p* < 0.001); those in the H/LB(+) group were higher than those in the M/LB(+) and L/LB(+) groups (*p* < 0.001); and those in the H/EA + H/LB(+) group showed the highest expression levels compared with the other groups (*p* < 0.001). After challenge, the chickens in the H/LB(+) group displayed less inflammatory injury than those in the M/LB(+) and L/LB(+) groups (*p* < 0.05), and the chickens in the H/EA + H/LB(+) group showed stronger anti-inflammatory effects than the other groups (*p* < 0.05). Thus, these protective effects against infection were consistent with the above results. Overall, significant anti-inflammatory effects were observed in chickens orally gavaged with high dosages of live *L. brevis* 23017 and ellagic acid, which occurred by regulation of the ChTLR15/NLRP3/IL-1β pathway.

## Introduction

Avian coccidiosis is an intestinal protozoan disease caused by at least seven *Eimeria* species. Chickens infected by *E. tenella*, the most pathogenic *Eimeria* parasite, showed severe clinical symptoms, including bloody diarrhoea and dehydration. Infection with *Eimeria* leads to a decline in feed utilization efficiency and body weight gain, therefore ultimately causing serious economic losses to the commercial poultry industry. Previous studies have focused on preventing coccidiosis from the view of preventive immunity using attenuated live vaccines [1] and on interfering with parasite development through the use of anticoccidial drugs [2]. Recently, studies on novel anticoccidial products have become a research hotspot, aiming to overcome the drawbacks of traditional drugs and live vaccines, such as the emergence of drug-resistant parasites and virulence reversion of live vaccines.

The life cycle of *Eimeria* parasites is complex and consists of sexual and asexual stages. The immune responses and detailed mechanisms stimulated by *Eimeria* in different stages has not been very clear until now. It has been reported that the elicited innate immune responses during infection with the protozoan *Toxoplasma gondii* display a close relationship with inflammatory injury [3]. Our previous study showed that the inflammatory pathways of chicken NOD-like receptor 3 (ChNLRP3) and chicken interleukin 1 beta (ChIL-1β) are strictly related to the activation of chicken Toll-like receptor 15 (ChTLR15), chicken myeloid differentiation primary response 88 (ChMyD88) and the chicken nuclear transcription factor-κB (ChNF-κB) pathway, which is specifically activated by *E. tenella* sporozoites [4]. The previous results suggested that drugs or biological products with the capability to inhibit the ChTLR15/NF-κB-ChNLRP3/IL-β pathway probably play a vital role in attenuating the inflammatory injury caused by *Eimeria* infection. Thus, an in-depth exploration of inhibitors of the ChTLR15/NF-κB-ChNLRP3/IL-β signalling pathway and the effects on the attenuation of intestinal inflammatory injury caused by *Eimeria* infection may be a promising strategy to develop new anticoccidial preparations.

Increasing evidence has revealed that lactic acid bacteria effectively activate nuclear factor erythroid 2-related factor 2 (Nrf2) antioxidant response elements (AREs), which further initiate the expression of serial antioxidant genes and exert antioxidant and anti-inflammatory effects [5–7]. Lin et al. reported that *Lactobacillus plantarum AR501*, isolated from Chinese food, markedly elevated the expression levels of Nrf2 and several antioxidant genes, including the glutathione S-transferase GSTO1, haem oxygenase-1 (HO-1), glutamate cysteine ligase (GCL), and NAD(P)H: quinone oxidoreductase-l (NQO1), in mouse livers [8], indicating that Nrf2/HO-1 is an important antioxidant signalling pathway. Another previous report demonstrated that *Lactobacillus brevis* 23017 effectively ameliorates intestinal inflammation and alleviates oxidative stress in animal models [9]. It was also reported that natural polyphenolic compounds, which are distributed extensively in medicinal plants, alleviate oxidative stress and inflammatory injury by upregulating the Nrf2 pathway [10, 11]. Ellagic acid (EA) is a polyphenolic compound that has been extracted from several vegetables, fruits and berries [12], has been extensively recognized to trigger the Nrf2 pathway, and displays antioxidant and anti-inflammatory effects [13]. Accumulating evidence reveals that the Nrf2 signalling pathway regulates the activation of the TLR/MyD88/NF-κB pathway, which further influences the activation of the NLRP3/IL-β pathway [14, 15]. Therefore, will EA and *L. brevis* 23017, two activators of the Nrf2 pathway, exert anticoccidial effects by inhibiting overactivation of the TLR/NLRP3/IL-β pathway? The aim of the present study was to explore whether oral administration of *L. brevis* 23017 alone and in combination with EA attenuated intestinal inflammatory injury caused by *E. tenella* infection by regulating the ChTLR15/NF-κB/NLRP3/IL-1β pathway.

## Materials and methods

### Chickens, parasites, bacteria and drugs

One-day-old specific-pathogen-free (SPF) Leghorn chickens were purchased from Harbin Veterinary Research Institute, China. *E. tenella* was stored in the Laboratory of Veterinary Pathology, Northeast Agricultural University, China, and propagated by challenging the chickens every six months to maintain pathogenicity. *L. brevis* 23017 was kindly provided by Professor Junwei Ge, Department of Preventive Veterinary Medicine, Northeast Agricultural University, China, and was reported to effectively ameliorate intestinal inflammation and alleviate oxidative stress in animal models [9]. EA was purchased from Shanghai Yuanye Biological Co., Ltd. (Shanghai, China).

### Design of animal experiments

Two animal experiments were designed and performed. The design for animal Experiment 1 is outlined in Table 1. Eleven-day-old chickens were randomly allocated into 7 groups with 15 chickens in each group. Each chicken in Group 1 was orally gavaged with 200 μL of PBS (pH 7.2) (PBS group). Each chicken in Groups 2–4 was orally gavaged with a low (5.0 × 10^8^ CFU in 200 μL of PBS), medium (5.0 × 10^9^ CFU in 200 μL of PBS) or high (5.0 × 10^10^ CFU in 200 μL of PBS) dose of live *L. brevis* 23017; these groups were named the L/LB(+), M/LB(+), and H/LB(+) groups, respectively. Each chicken in Groups 5–7 was orally gavaged with a low (5.0 × 10^8^ CFU in 200 μL of PBS), medium (5.0 × 10^9^ CFU in 200 μL of PBS) or high (5.0 × 10^10^ CFU in 200 μL of PBS) concentration of heat-killed *L. brevis* 23017; these groups were called the L/LB(−), M/LB(−), and H/LB(−) groups, respectively. From 11 to 20 days of age, all chickens in each group in Experiment 1 were orally gavaged once a day.

**Table 1:** **Design of animal experiments**

Group		Dosage per chicken			Number of chickens	Design of animal experiment
Experiment 1						
1	PBS (pH 7.2)			200 µL	15	Chickens were orally gavaged with PBS, LB(+) and LB(−) once a day from 11 to 20 days of age
2	L/LB(+)	5.0 × 10^8^ CFU		200 µL	15	
3	M/LB(+)	5.0 × 10^9^ CFU			15	
4	H/LB(+)	5.0 × 10^10^ CFU			15	
5	L/LB(−)	5.0 × 10^8^ CFU		200 µL	15	
6	M/LB(−)	5.0 × 10^9^ CFU			15	
7	H/LB(−)	5.0 × 10^10^ CFU			15	
Experiment 2						
1	PBS (pH 7.2)		200 µL		15	Chickens were gavaged with LB(+) once a day from 11 to 20 days of age, which was combined with EA from 16 to 20 days of age. All chickens except those in the PBS group were challenged with 50 000 *E. tenella* sporulated oocysts at 21 days of age
2	L/EA + L/LB(+)	15.0 mg/kg + 5.0 × 10^8^ CFU	200 µL		15	
3	M/EA + M/LB(+)	30.0 mg/kg + 5.0 × 10^9^ CFU			15	
4	H/EA + H/LB(+)	60.0 mg/kg + 5.0 × 10^10^ CFU			15	
5	Challenge control	/	/		15	

For Experiment 1, each chicken in Group 1 was orally gavaged with PBS (pH 7.2), and those in Groups 2–4 were orally gavaged with a high (5.0 × 10^10^ CFU in 100 μL of PBS), medium (5.0 × 10^9^ CFU in 100 μL of PBS) or low dose (5.0 × 10^8^ CFU in 100 μL of PBS) of live *L. brevis* 23017; these groups are abbreviated as the H/LB(+), M/LB(+), and L/LB(+) groups, respectively. Groups 5–7 were orally gavaged with heat-killed bacteria *L. brevis* 23017 at a high (5.0 × 10^10^ CFU in 100 μL of PBS), medium (5.0 × 10^9^ CFU in 100 μL of PBS) or low dose (5.0 × 10^8^ CFU in 100 μL of PBS); these groups are abbreviated as H/LB(−), M/LB(−), and L/LB(−), respectively.

For Experiment 2, each chicken in Group 1 was orally gavaged with PBS (pH 7.2), and those in Groups 2–4 were orally gavaged with one of three dosages of LB(+) once a day from 11 to 20 days of age. Additionally, three dosages of EA were administered from 16 to 20 days of age. Group 5 was the control challenge group. All chickens except those in the PBS group were challenged with 50 000 *E. tenella* sporulated oocysts at 21 days of age.

The design for Experiment 2 is outlined in Table 1. Sixteen-day-old chickens were randomly divided into 5 groups with 15 chickens in each group. Each chicken in Group 1 was orally gavaged with 200 μL of PBS (pH 7.2) (PBS group). The chickens in Groups 2–4 were orally gavaged with L/LB(+), M/LB(+), or H/LB(+) once a day from 11 to 20 days of age combined with a low (15 mg/kg), medium (30 mg/kg) or high (60 mg/kg) dosage of EA once a day from 16 to 20 days of age; these groups were named the L/EA + L/LB(+), M/EA + M/LB(+), H/EA + H/LB(+) groups, respectively. Group 5 was designated as the challenged control group. At 21 days of age, all of the chickens in Experiments 1 and 2, except those in the PBS groups (nonchallenged control group), were orally gavaged with 50 000 *E. tenella* sporulated oocysts. Animal experiments were performed according to the regulations of the Ethics Committee for Animal Sciences at Northeast Agricultural University, Heilongjiang Province, China (NEAUEC20210332).

### RNA extraction from the caeca

Chickens randomly selected from each group (*n* = 8) were euthanized, and the whole caecal tissue from each chicken was harvested for RNA extraction. Total RNA extraction was carried out using a purification kit (Sigma–Aldrich), and cDNA was synthesized as described in our previous report [4].

### Real-time PCR

Real-time PCR (qRT–PCR) was performed using SYBR^®^ Premix Ex Taq™ II (Tli RNase H Plus) (TaKaRa Biotech Corp., Dalian, China) according to the manufacturer’s instructions. qRT–PCR was carried out on a LightCycler 480 (Roche) according to the minimum information for publication of qRT–PCR experiments (MIQE) guidelines [16]. The mRNA expression levels of chicken glyceraldehyde-3-phosphate dehydrogenase (GAPDH) in the caecum were shown to be stable in the preliminary test and GAPDH was therefore selected as an internal reference gene. The primer pairs in this study were designed according to the target gene sequences from GenBank using Oligo 6.0 software and are shown in Table 2. When the amplification efficiencies of the 100-fold serially diluted target and reference gene cDNA samples were similar, the 2^−ΔΔCt^ method was used to quantify the target gene [17].

**Table 2:** **Primer sequences used for real-time PCR**

Genes and GenBank accession numbers	Primer sequence (5′–3′)	Fragment size (base pairs)
Nrf2 (MN416129.1)	AGCAGTGAATAGCAACACCAGTCC CGAGGCTGCTGTCTGTATCTGAAG	125
HO-1 (X56201.1)	GATGCGTTCTGGCGGTGCTC GCTGTCGGTGCTGTTGCTCTG	147
GCLC (XM_419910.5)	GGAGAGGCGGTGTAAGAGAAGAGG GACTGTGGGAGCAGCAGCAATG	137
GCLM (NM_001007953.1)	GCTGCTAACTCACAATGACC TGCATGATATAGCCTTTGGAC	127
GPx1 (NM_001277853.2)	GACCAACCCGCAGTACATCA GAGGTGCGGGCTTTCCTTTA	112
NQO1 (NM_001277621.1)	AAGGGCTGGGAAGTCACCAT CGTAGACAAAGCACTCGGGG	107
ChTLR15 (FJ915250.1)	GGCTGTGGTATGTGAGAATG ATCGTGCTCGCTGTATGA	155
MyD88 (NM_001030962.4)	GCCTCGGCCTTTACCTCAAC CCGGATCTCCAGGTAGTCGT	92
NF-κB (M86930.1)	TCTGAACAGCAAGTCATCCATAACG AAGGAAGTGAGGTTGAGGAGTCG	127
NLRP3 (KF318520.1)	GGTTTACCAGGGGAAATGAGG TTGTGCTTCCAGATGCCGT	114
IL-10 (AJ621254.1)	CAGCACCAGTCATCAGCAGCAGAGC GCAGGTGAAGAAGCGGTGACAG	94
IL-18 (GU119895.1)	ATCGCAGTGTGTGCAGTACG ACGAACCACAAGCAACTGGC	141
IL-1β (HQ739080.1)	CAGTGTGTGCAGTACGGCTT ACGCTGAATGCAACAGGCAT	268
GAPDH (JQ280469.1)	GACGTGCAGCAGGAACACTA ATGGCCACCACTTGGACTTT	129

### Enzyme-linked immunosorbent assay

Two grams of caecal tissue from each chicken was homogenized in 2 mL of saline with a high-speed tissue homogenizer (Kinematica, Switzerland). Then, the concentrations of Nrf2, superoxide dismutase (SOD), glutathione (GSH) and catalase (CAT) in the caecal homogenates from the different groups in Experiments 1 and 2 were determined by using enzyme-linked immunosorbent assay (ELISA) kits (Enzyme-linked Biotechnology Co. Ltd., Shanghai, China). Operations were carried out according to the manufacturer’s instructions. Concentrations were calculated from standard curves.

### Levels of in the caeca prior to challenge

The mRNA expression levels of Nrf2, HO-1, glutamyl-cysteine synthetase catalytic subunit (GCLC), glutamate-cysteine ligase modified subunit (GCLM), glutathione peroxidase-1 (GPx1), and NQO1 in the caeca of the chickens (*n* = 8) from different groups in Experiments 1 and 2, including three live *L. brevis* 23017 groups, three heat-killed *L. brevis* 23017 groups, and three EA combined with live *L. brevis* 23017 groups, were quantified using qRT–PCR at 21 days of age. The concentrations of Nrf2, SOD, GSH and CAT in the caeca of the chickens (*n* = 8) from each group were determined by ELISA.

### Levels of ChTLR15/ChNLRP3/ChIL-1β pathway components in the caeca post-challenge

On Day 7 post-infection (pi), the caeca of the chickens (*n* = 7) from the different groups in Experiments 1 and 2, including three live *L. brevis* 23017 groups, three heat-killed *L. brevis* 23017 groups, and three EA combined with live *L. brevis* 23017 groups, were sampled for quantification of the mRNA expression levels of ChTLR15, ChMyD88, ChNF-κB, ChNLRP3, chicken cysteinyl aspartate specific proteinase 1 (ChCaspase-1), ChIL-1β, chicken interleukin 10 (ChIL-10) and ChIL-18 using qRT–PCR. The concentrations of ChTLR15, ChNLRP3, and ChIL-1β in the caeca of the chickens (*n* = 7) from each group were determined by ELISA.

### Oxidant enzyme levels in the caeca post-challenge

The contents of malondialdehyde (MDA), a product of oxidative stress, in the caeca of the chickens (*n* = 7) from each group in Experiments 1 and 2 were detected using ELISA.

### Anticoccidial effects

Chickens from each group in Experiments 1 and 2 were weighed at 21 days of age (before challenge) and at 28 days of age (on Day 7 post-challenge) to calculate the body weight gain (BWG) as previously described [18]. At 7 days post-challenge, caecal samples of the chickens (*n* = 7) from each group were harvested for gut lesion scoring based on the method described in a previous report [19]. Faecal samples from chickens housed separately within each group between Days 7 and 11 post-challenge were gathered, and oocyst counting was performed microscopically by three different scientists using the McMaster counting technique as described in a published report [18]. The oocyst reduction ratio was determined using the following formula: oocyst reduction ratio = (number of oocysts from chickens in the challenged control group-number of oocysts from the other groups)/number of chickens in the challenged control group × 100%.

### Pathological changes in the caeca

On Day 7 pi, the caecum of each chicken from each group in Experiment 2 (*n* = 7) was sampled for gross pathological observation. The caecal samples were fixed in neutral buffered formalin (10%), embedded in paraffin, sectioned at 4 μm, and stained with haematoxylin and eosin (HE). The histopathological lesions in the caecal tissues were observed using a light microscope (Nikon EX200).

### Statistical analysis

Data are expressed as the means ± standard deviation (SD) and subjected to one-way analysis of variance (ANOVA) with Tukey’s multiple-comparison procedures with GraphPad Prism 5 software, and the differences between the mean values were analysed. Differences were considered significant at *p* < 0.05.

## Results

### mRNA expression levels of Nrf2/HO-1 pathway components in the caeca prior to challenge

**Figure 1:**
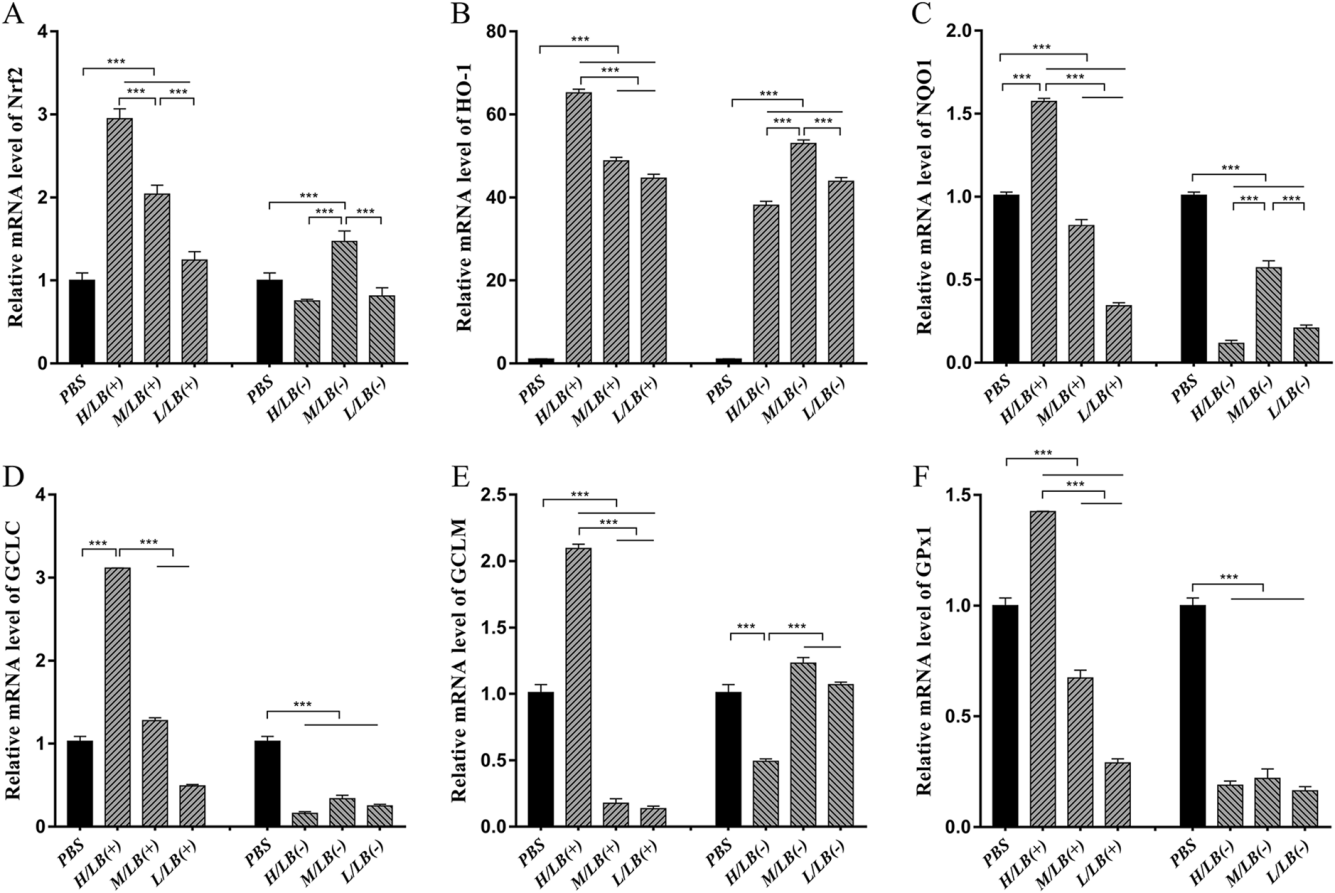
**The mRNA levels of the components of the Nrf2/HO-1 pathway found in the caeca of chickens from the different groups in Experiment 1 prior to challenge.** The mRNA levels of **A** Nrf2, **B** HO-1, **C** NQO1, **D** GCLC, **E** GCLM and **F** GPx1 in the caeca of chickens (*n* = 8) in each group in Experiment 1 were examined by real-time PCR. Three dosages (low, medium and high) of live and heat-killed *L. brevis* 23017 were used to establish the groups named L/LB(+), M/LB(+) and H/LB(+), and L/LB(−), M/LB(−) and H/LB(−), respectively. GAPDH was selected as an internal reference gene, and the 2^−ΔΔCt^ method was applied to quantify the target gene. Data are shown as the mean ± SD. ***p < 0.001.

Prior to oral challenge with *E. tenella*, the mRNA expression levels of Nrf2, HO-1, GCLC, GCLM, GPx1 and NQO1 in the caecal tissues of the chickens from the LB(+) groups were all significantly higher than those in the LB(−) groups (Figure 1) (*p* < 0.001). Among the three LB(+) groups, the mRNA levels of all target genes in the H/LB(+) group were higher than those in the M/LB(+) and L/LB(+) groups (Figure 1) (*p* < 0.001).

**Figure 2:**
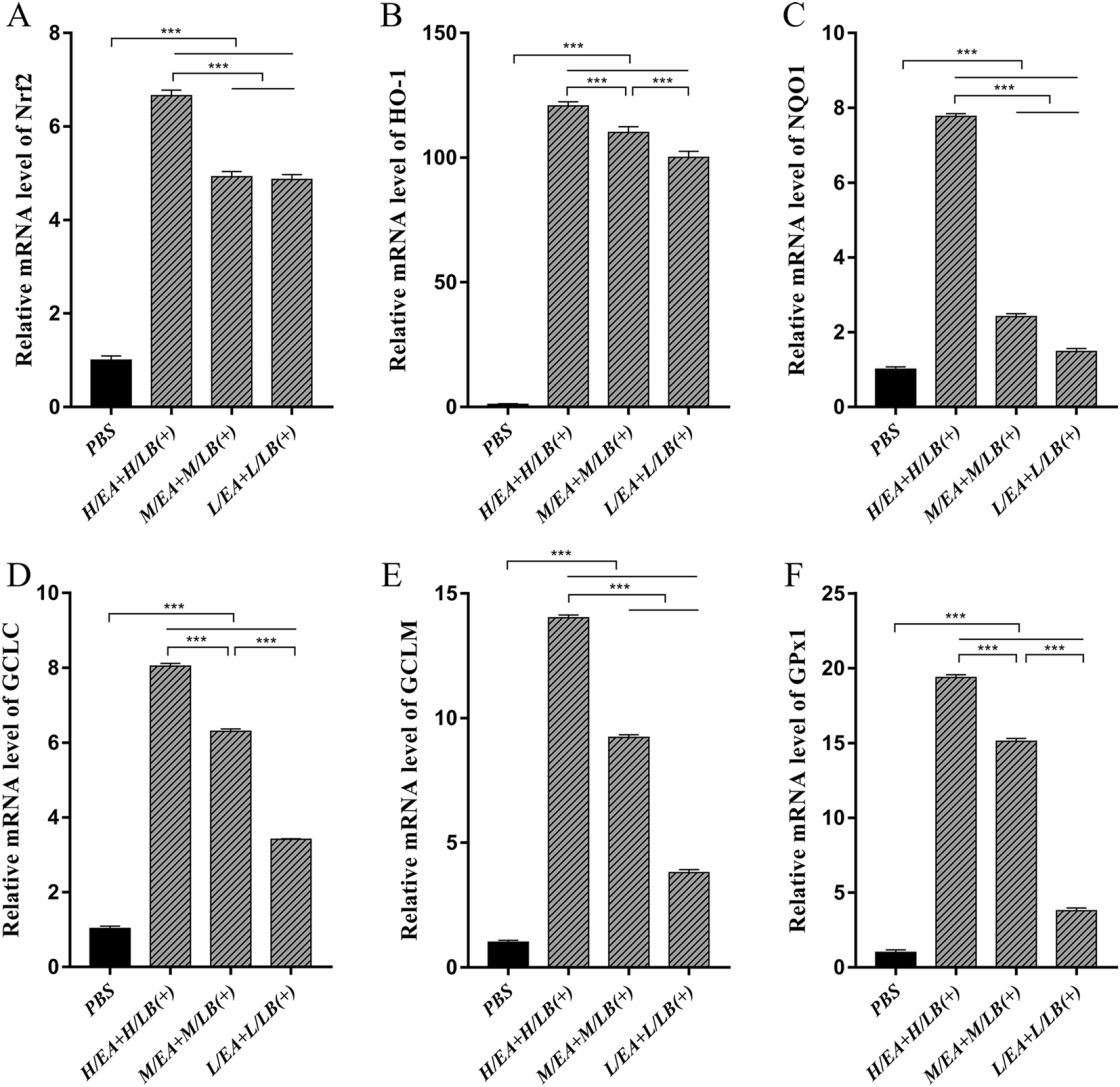
**The mRNA levels of the components of the Nrf2/HO-1 pathway found in the caeca of chickens from the different groups in Experiment 2 prior to challenge.** The mRNA levels of **A** Nrf2, **B** HO-1, **C** NQO1, **D** GCLC, **E** GCLM and **F** GPx1 in the caeca of chickens (*n* = 8) in each group in Experiment 2 were quantified by real-time PCR. The groups were gavaged with low, medium and high dosages of EA and combined with L/LB(+), M/LB(+), and H/LB(+) to give groups named L/EA + L/LB(+), M/EA + M/LB(+), and H/EA + H/LB(+), respectively. Data are shown as the mean ± SD. ***p < 0.001.

To explore whether the combination of LB(+) and EA provided higher antioxidant levels in the caeca, the chickens in Experiment 2 were gavaged with H/LB(+), M/LB(+) and L/LB(+) from 11 to 20 days of age and then with H/EA, M/EA and L/EA, respectively, from 16 to 20 days of age. The results showed that the chickens orally administered H/EA + H/LB(+) showed the strongest antioxidant levels in the caeca compared with chickens in the other groups (*p* < 0.001) (Figure 2).

### Protein levels of antioxidant enzymes in the caeca prior to challenge

**Figure 3:**
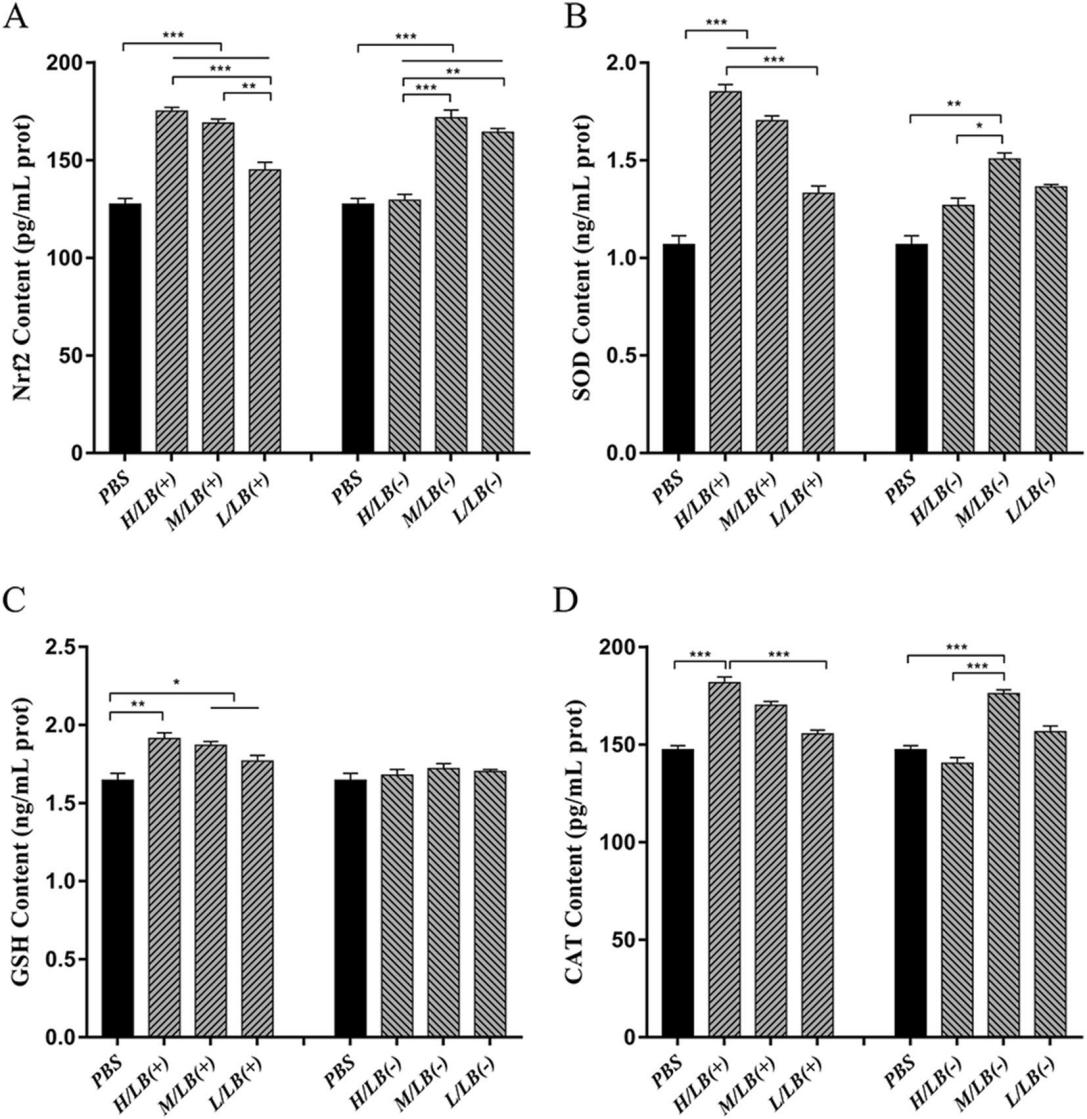
**Protein levels of Nrf2, SOD, GSH and CAT in the caeca of chickens in the different groups in Experiment 1 prior to challenge.** The protein expression levels of **A** Nrf2, **B** SOD, **C** GSH and **D** CAT in the caeca of chickens (*n* = 8) in each group in Experiment 1 were determined by ELISA. Three dosages (low, medium and high) of live and heat-killed *L. brevis* 23017 were used to establish the groups named L/LB(+), M/LB(+) and H/LB(+), and L/LB(−), M/LB(−) and H/LB(−), respectively. Data are shown as the mean ± SD. *p < 0.05, **p < 0.01, ***p < 0.001.

**Figure 4:**
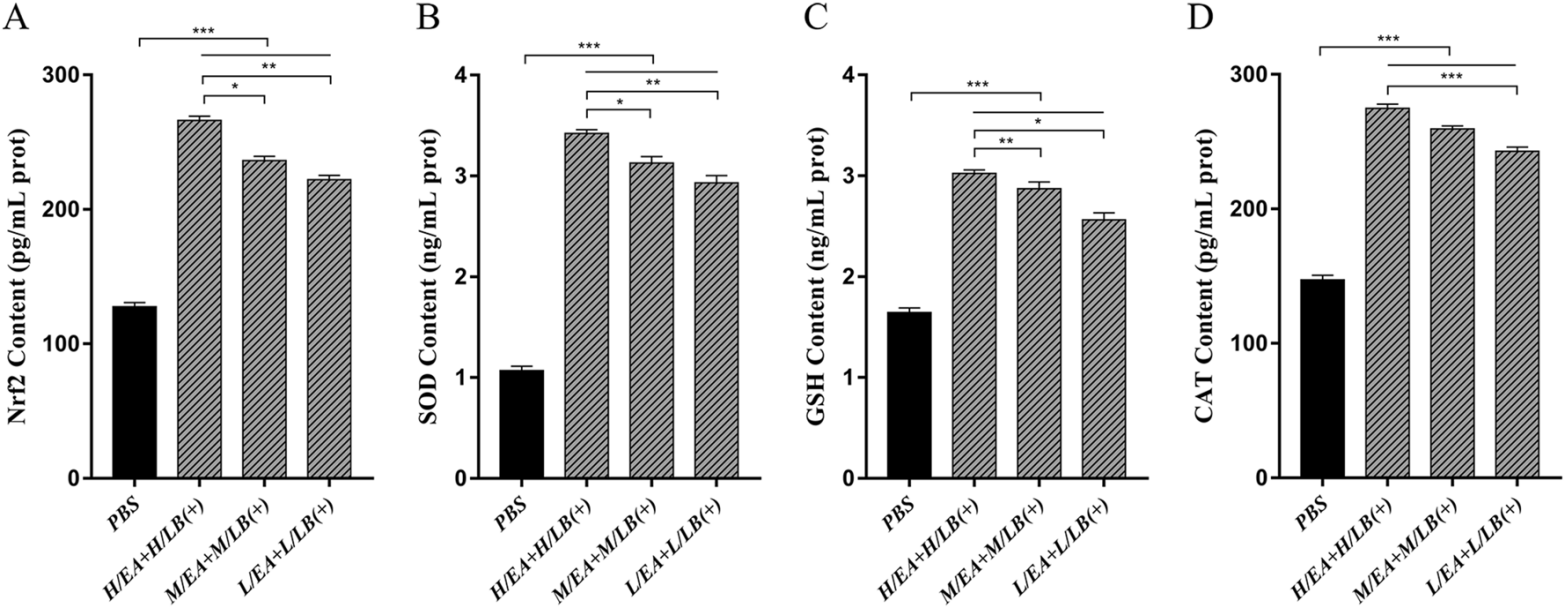
**Protein levels of Nrf2, SOD, GSH and CAT in the caeca of chickens in the different groups in Experiment 2 prior to challenge.** The protein expression levels of **A** Nrf2, **B** SOD, **C** GSH and **D** CAT in the caeca of chickens (*n* = 8) in each group in Experiment 2 were determined by ELISA. EA was combined with live *L. brevis* 23017 at three different dosages, and these groups were named L/EA + L/LB(+), M/EA + M/LB(+) and H/EA + H/LB(+). Data are shown as the mean ± SD. *p < 0.05, **p < 0.01, ***p < 0.001.

Prior to challenge, the expression levels of antioxidant enzymes, including Nrf2, SOD, GSH and CAT, in the caeca of the chickens in the LB(+) and LB(−) groups, and in particular, the H/LB(+) group, were significantly upregulated compared with those in the PBS group (*p* < 0.01) (Figure 3). Notably, the H/EA + H/LB(+) group displayed the highest levels of antioxidant enzymes compared with the M/EA + M/LB(+), L/EA + M/LB(+) and control groups (*p* < 0.001) (Figure 4).

### Levels of ChTLR15/ChNLRP3 pathway components in the caeca post-challenge

**Figure 5:**
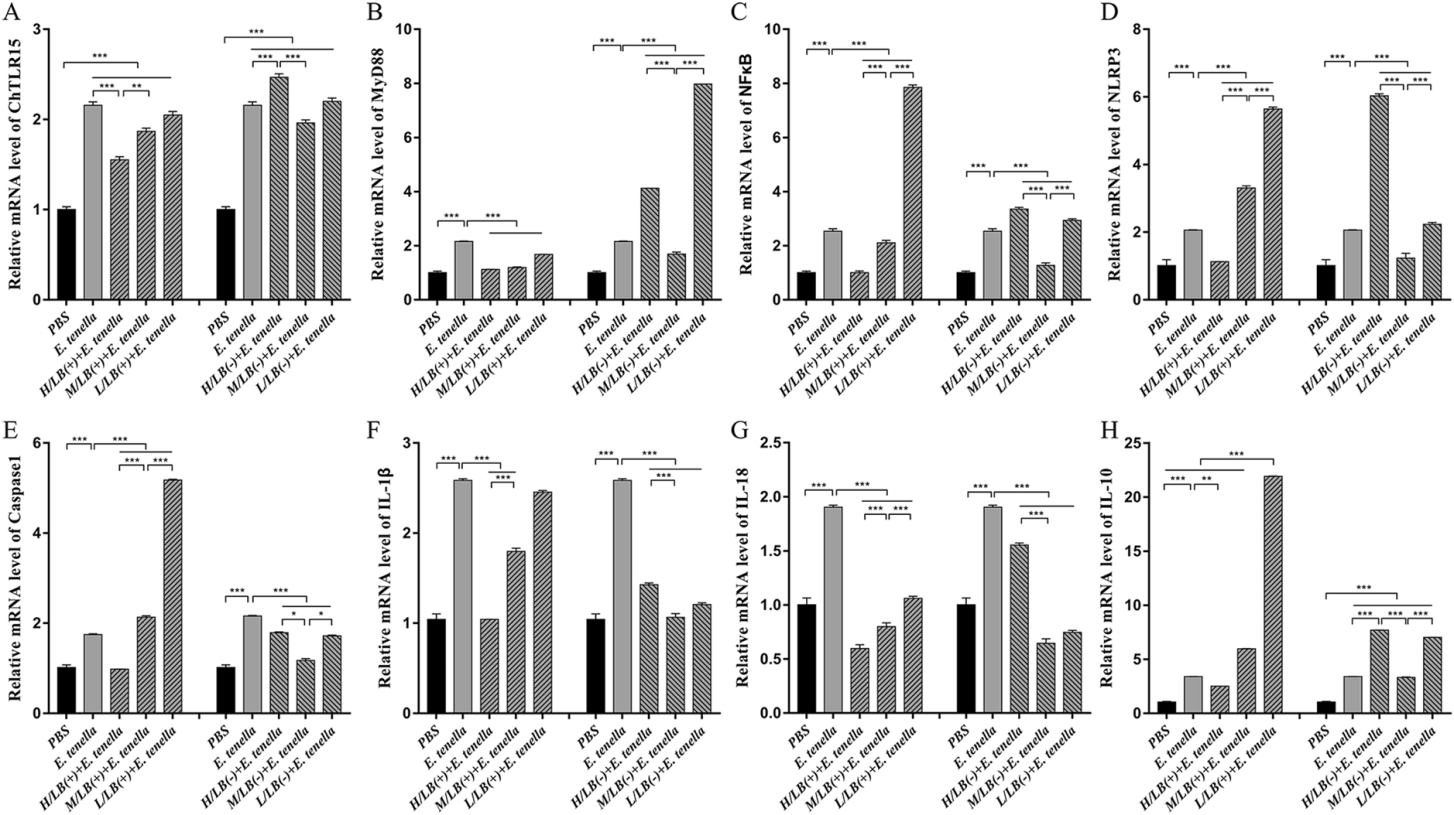
**The mRNA levels of the components of the ChTLR15/ChNLRP3/ChIL-1β pathway in the caeca of chickens from the different groups in Experiment 1 post-challenge with *E. tenella*.** On Day 7 pi with *E. tenella*, the caeca of chickens (*n* = 7) from each group in Experiment 1 were sampled for quantification of the mRNA expression levels of **A** ChTLR15, **B** ChMyD88, **C** ChNF-κB, **D** ChNLRP3, **E** ChCaspase-1, **F** ChIL-1β, **G** ChIL-18 and **H** ChIL-10 using real-time PCR. Three dosages (low, medium and high) of live and heat-killed *L. brevis* 23017 were established to give the groups named L/LB(+), M/LB(+) and H/LB(+), and L/LB(−), M/LB(−) and H/LB(−), respectively. Data are shown as the mean ± SD. ***p < 0.001.

**Figure 6:**
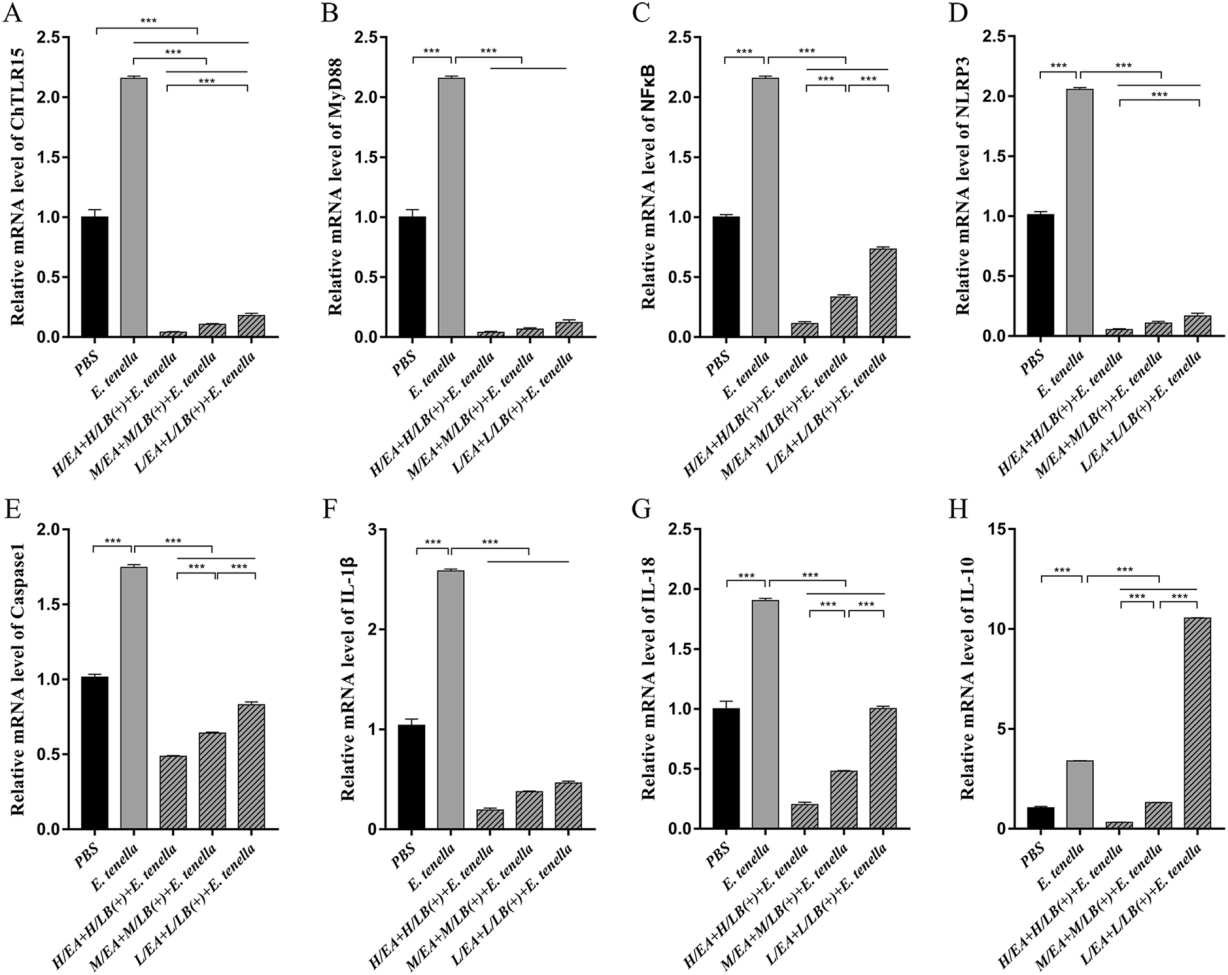
**The mRNA levels of in the components of the ChTLR15/ChNLRP3/ChIL-1β pathway in the caeca of *E. tenella*-infected chickens from the different groups in Experiment 2.** On Day 7 pi with *E. tenella*, the caeca of chickens (*n* = 7) from each group in Experiment 2 were sampled, and the mRNA expression levels of **A** ChTLR15, **B** ChMyD88, **C** ChNF-κB, **D** ChNLRP3, **E** ChCaspase-1, **F** ChIL-1β, **G** ChIL-18 and **H** ChIL-10 were quantified by real-time PCR. EA was combined with live *L. brevis* 23017 at three different dosages, and the groups were named L/EA + L/LB(+), M/EA + M/LB(+) and H/EA + H/LB(+). Data are shown as the mean ± SD. ***p < 0.001.

**Figure 7:**
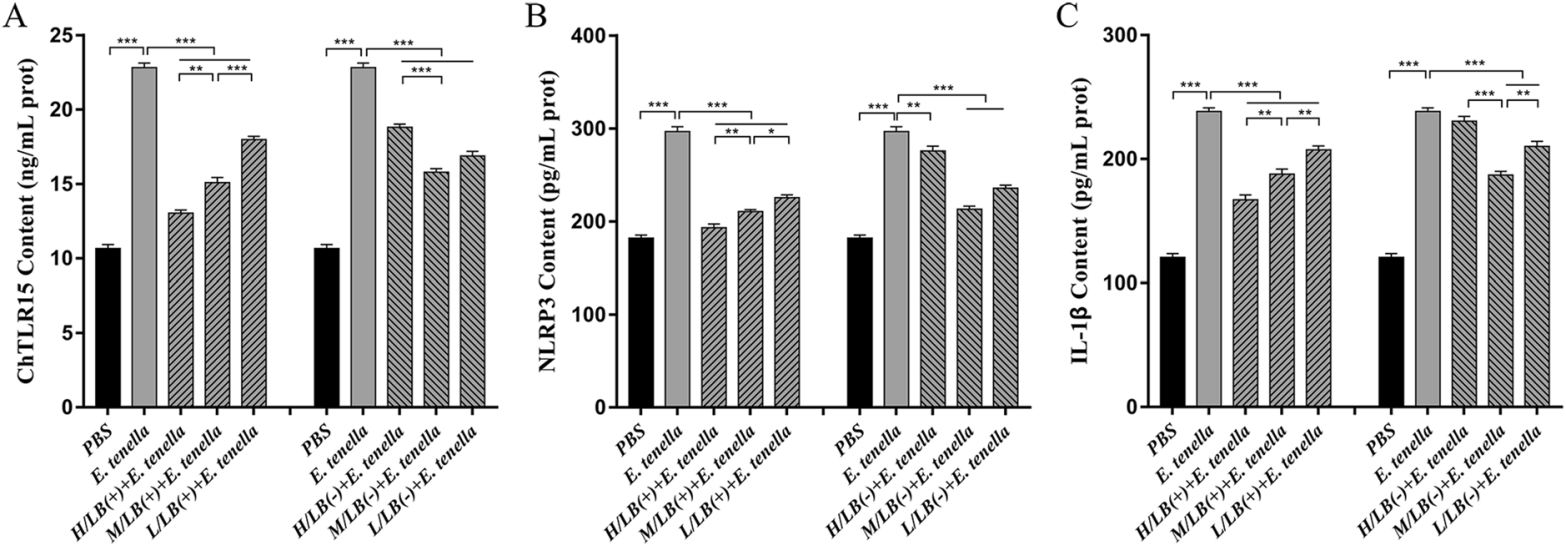
**Protein levels of ChTLR15, NLRP3 and ChIL-1β in the caeca of chickens from the different groups in Experiment 1 post-challenge with *E. tenella*.** On Day 7 pi, the protein expression levels of **A** ChTLR15, **B** NLRP3 and **C** ChIL-1β in the caeca of each group of *E. tenella*-challenged chickens (*n* = 7) in Experiment 1 were determined by ELISA. The groups receiving low, medium and high dosages of live and heat-killed *L. brevis* 23017 were named L/LB(+), M/LB(+) and H/LB(+), and L/LB(−), M/LB(−) and H/LB(−), respectively. Data are shown as the mean ± SD. **p < 0.01, ***p < 0.001.

**Figure 8:**
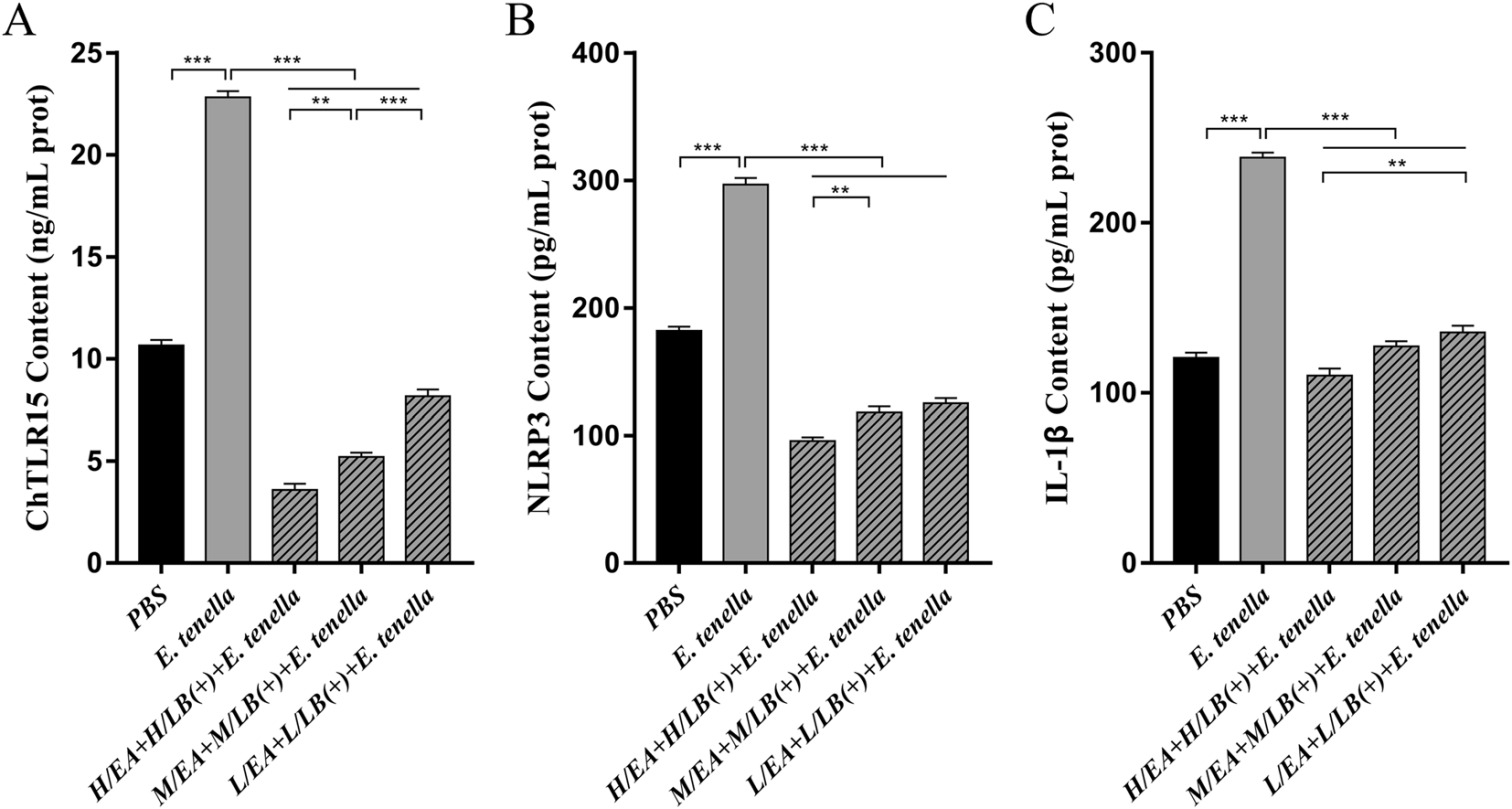
**Protein levels of ChTLR15, NLRP3 and ChIL-1β in the caeca of chickens from the different groups in Experiment 2 post-challenge with *E. tenella*.** On Day 7 pi, the protein expression levels of **A** ChTLR15, **B** NLRP3 and **C** ChIL-1β in the caeca of each group of *E. tenella*-challenged chickens (*n* = 7) in Experiment 2 were determined by ELISA. EA was combined with live *L. brevis* 23017 at three different dosages, and the groups were named L/EA + L/LB(+), M/EA + M/LB(+) and H/EA + H/LB(+). Data are shown as the mean ± SD. **p < 0.01, ***p < 0.001.

After challenge with *E. tenella* sporulated oocysts, the mRNA expression levels of ChTLR15, ChMyD88, ChNF-κB, ChNLRP3, ChCaspase-1, ChIL-18 and ChIL-1β in the caeca of the chickens from the H/LB(+) (Figure 5) and H/EA + H/LB(+) (Figure 6) groups were significantly downregulated compared with the *E. tenella*-challenged control group (*p* < 0.001). The protein levels of ChTLR15, ChNLRP3 and ChIL-1β in the caeca from the H/LB(+) (Figure 7) and H/EA + H/LB(+) (Figure 8) groups displayed the same change trend as the mRNA levels.

### Levels of the oxidant enzyme malondialdehyde (MDA) in the caeca post-challenge

**Figure 9:**
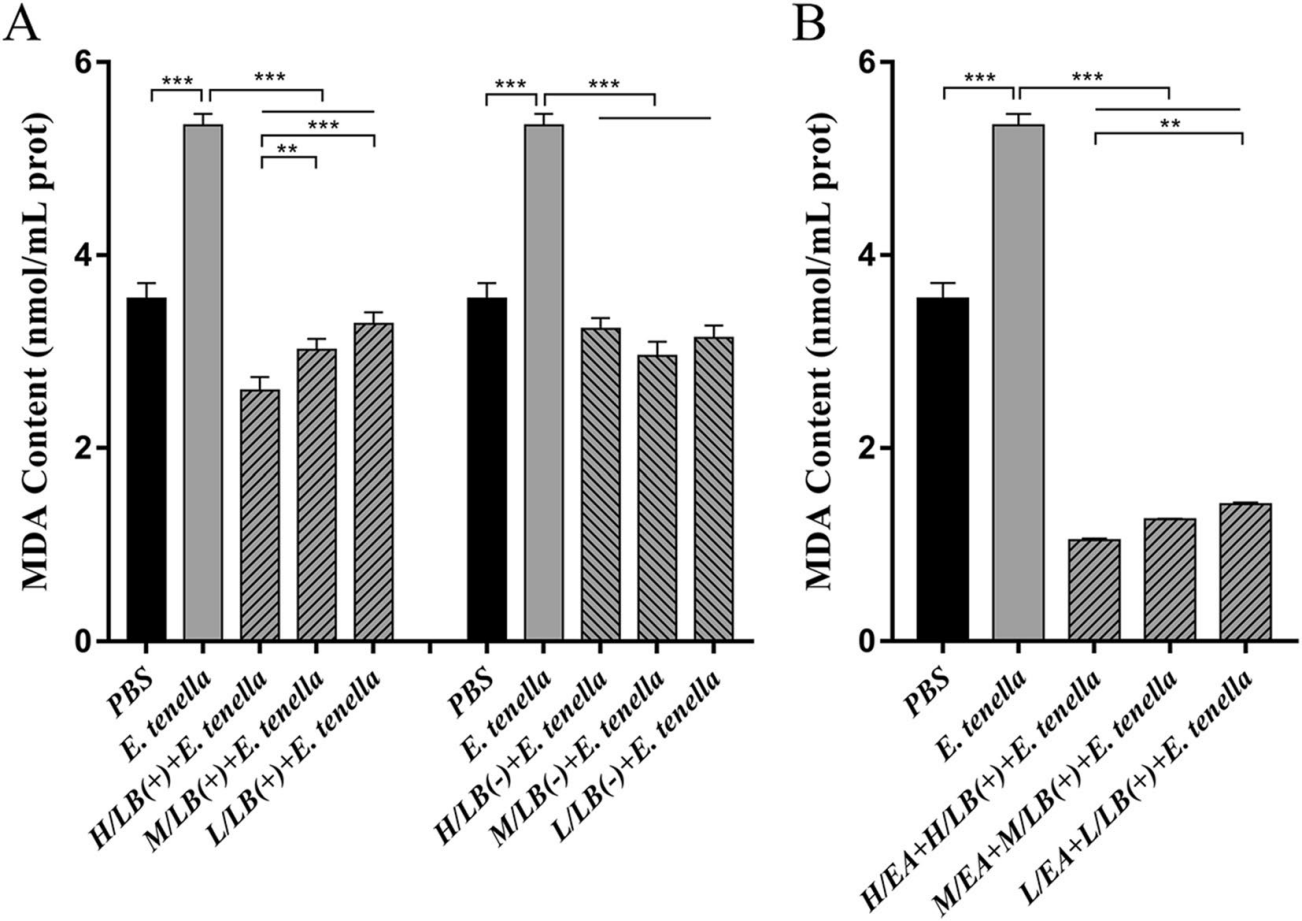
**Protein levels of the oxidant enzyme malondialdehyde (MDA) in the caeca of chickens from the different groups in Experiments 1 and 2 post-challenge with *E. tenella*.** The protein expression levels of MDA in the caeca of chickens (*n* = 7) in each group in Experiments 1 (**A**) and 2 (**B**) were determined by ELISA. Data are shown as the mean ± SD. **p < 0.01, ***p < 0.001.

The levels of the oxidant enzyme malondialdehyde (MDA) in the caeca of *E. tenella*-infected chickens were significantly higher than those of all the gavaged and challenged groups (Figure 9) (*p* < 0.001). After challenge, the level of MDA in the caeca of chickens from the H/LB(+) group was significantly downregulated compared with both the M/LB(+) and L/LB(+) groups (*p* < 0.001). The MDA levels in the EA combined with LB(+) groups were lower than those in the LB(+) groups, but statistically significant differences were not observed among the three EA + LB(+) groups (*p* > 0.05) (Figure 9).

### Evaluation of anticoccidial effects

**Figure 10:**
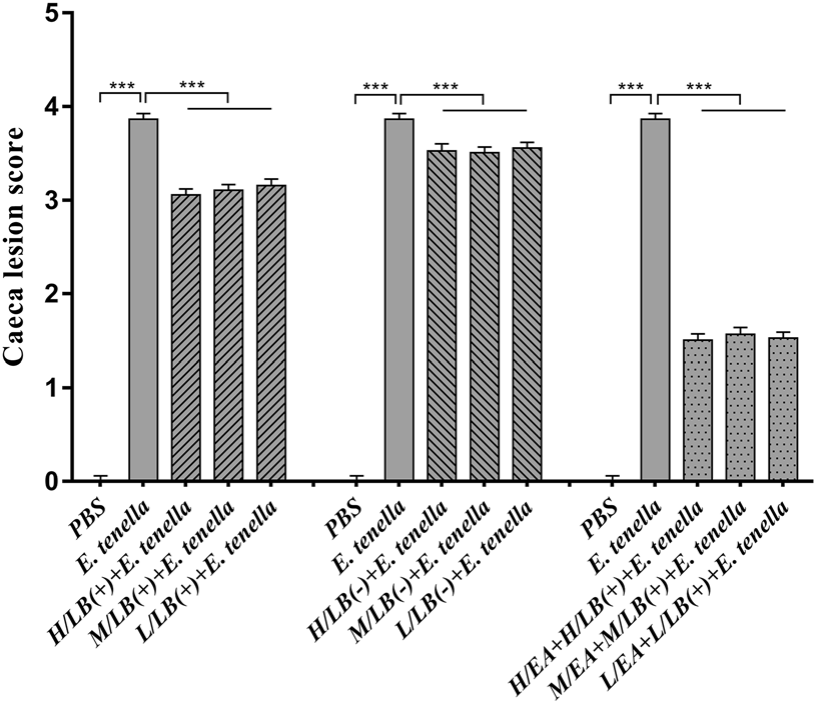
**Caecal lesion scores of the chickens in different groups in Experiments 1 and 2 post-challenge with *E. tenella*.** At 7 days post-challenge, caecal samples from each group of chickens (*n* = 7) in Experiments 1 and 2 were harvested for gut lesion scoring based on the method described by Johnson and Reid [19]. Data are shown as the mean ± SD. ***p < 0.001.

**Figure 11:**
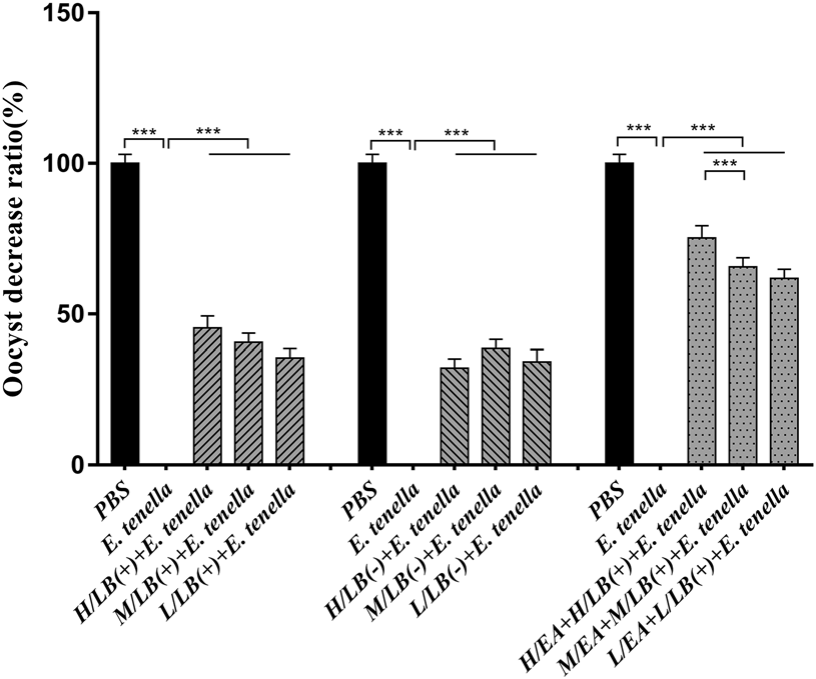
**The decrease in oocyst ratio in the different groups in Experiments 1 and 2 post-challenge with *E. tenella*.** Faecal samples chickens in each group raised in separate cages were gathered between Days 7 and 11 post-challenge. Oocyst counting was performed microscopically as described by Ma et al. [18], and the oocyst reduction ratio was determined. Data are shown as the mean ± SD. ***p < 0.001.

No chickens died from *E. tenella* infection. The average body weights of the chickens in the PBS group were higher than those in the other groups (*p* < 0.05). The H/EA + H/LB(+) group displayed the best effects on weight gain after *E. tenella* challenge, but a statistically significant difference was not observed compared with all of the LB(+), LB(−) and EA combined with LB(+) groups [*p* > 0.05 (Table 3)]. The average lesion scores in the caeca of the chickens in each treatment group were significantly lower than that in the *E. tenella-*infected control group (*p* < 0.01). Moreover, EA combined with LB(+), and especially H/EA combined with H/LB(+), displayed the lowest caecal lesion score compared with the other groups (*p* < 0.05) (Figure 10). The decreasing oocyst ratio from the chickens in each treatment group showed consistent changes in the caecal lesion scores and weight gain (Figure 11).

**Table 3:** **Body weight gain of the chickens in each group after challenge with** ***E. tenella***

Group	Survival rate (%)	Average body weight gain (g)	Relative body weight gain (%)
PBS	100	78.75 ± 2.46^a^	100
Experiment 1			
H/LB(+)	100	56.57 ± 3.85^b^	71.83
M/LB(+)	100	53.68 ± 3.85^b^	68.17
L/LB(+)	100	51.33 ± 3.27^b^	65.18
H/LB(−) + *E. tenella*	100	51.73 ± 3.27^b^	65.69
M/LB(−) + *E. tenella*	100	52.08 ± 2.94^b^	66.13
L/LB(−) + *E. tenella*	100	51.67 ± 3.85^b^	65.61
Experiment 2			
H/EA + H/LB(+)	100	66.46 ± 3.85^b^	84.39
M/EA + M/LB(+)	100	58.35 ± 3.85^b^	74.10
L/EA + L/LB(+)	100	56.48 ± 3.85^b^	71.72
Challenge control group	100	40.05 ± 2.65^c^	50.86

Data represent the mean ± SD. Significant differences (*p* < 0.05) between two numbers in a column are indicated by different lowercase letters. After oral administration of *L. brevis* 23017 combined with EA, all chickens except those in the PBS group were challenged with *E. tenella* sporulated oocysts at 21 days of age.

### Pathological changes in the caeca of chickens

**Figure 12:**
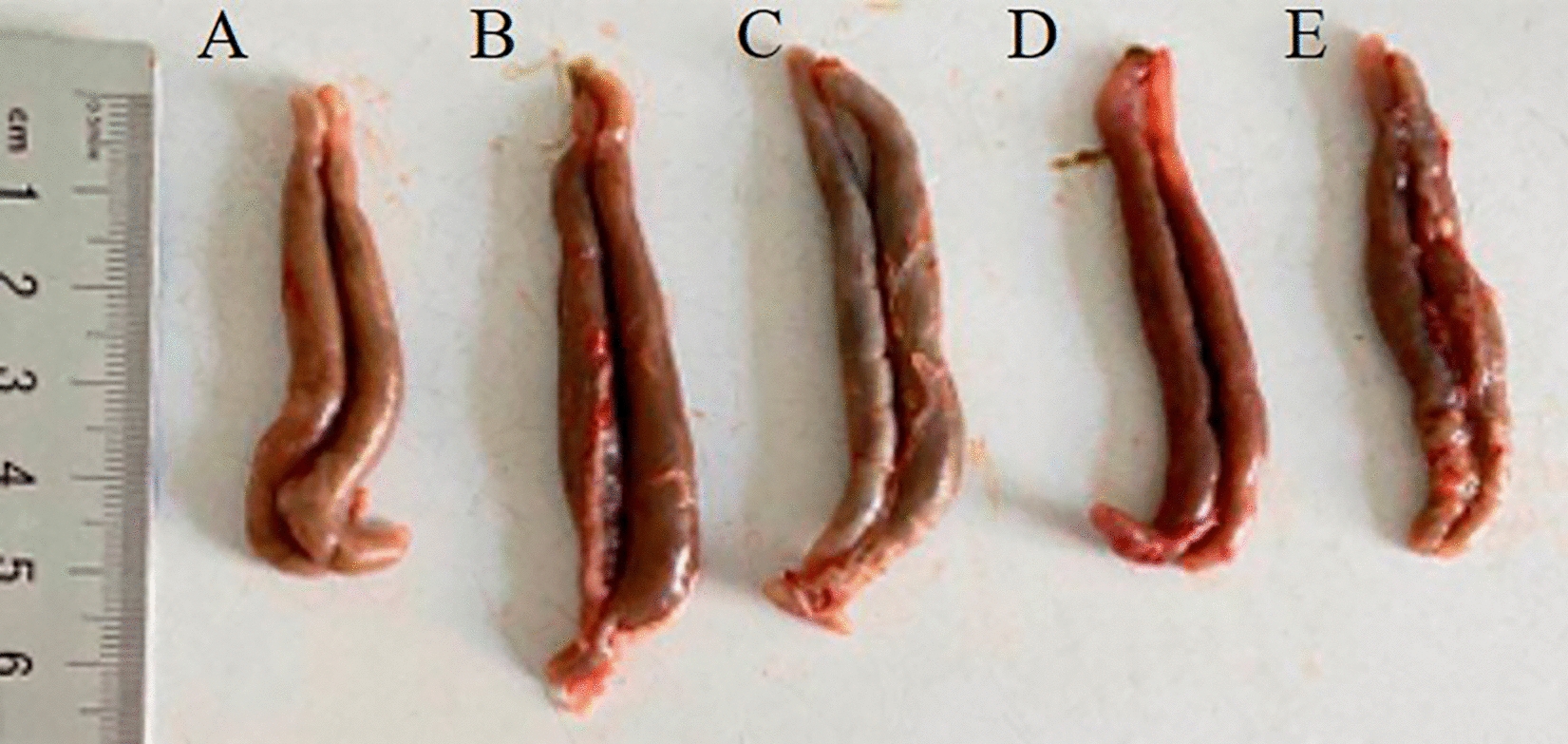
**Gross pathological changes in the caeca of chickens from the different groups in Experiment 2 post-challenge with *E. tenella*.** **A** The caeca of chickens in the PBS group showed no pathological changes. **B** On Day 7 post-challenge, the gross pathological changes in the caeca of chickens in the *E. tenella*-infected control group were remarkable and included swelling and haemorrhagic points on the surface of the caeca. However, the caeca of the chickens orally gavaged with the three different dosages (high, medium and low) of EA combined with live *L. brevis* 23017, the **C** L/EA + L/LB(+), **D** M/EA + M/LB(+)and **E** H/EA + H/LB(+)groups, and especially the H/EA + H/LB(+) group, displayed slight gross pathological changes.

On Day 7 post-challenge, no obvious gross pathological changes in the caeca of the chickens in the PBS group were observed (Figure 12A). Gross pathological changes in the caeca of the chickens in the *E. tenella*-infected control group (Figure 12B) were remarkable, including notable swelling, haemorrhagic points on the surface of the caeca, and a thickened intestinal wall. The caeca of the chickens in the L/EA + L/LB(+), M/EA + M/LB(+) and H/EA + H/LB(+) groups, and especially the H/EA + H/LB(+) group, displayed slight gross pathological changes (Figures 12C–E). The results indicated that EA combined with LB(+) displayed the most significant anticoccidial effects.

**Figure 13:**
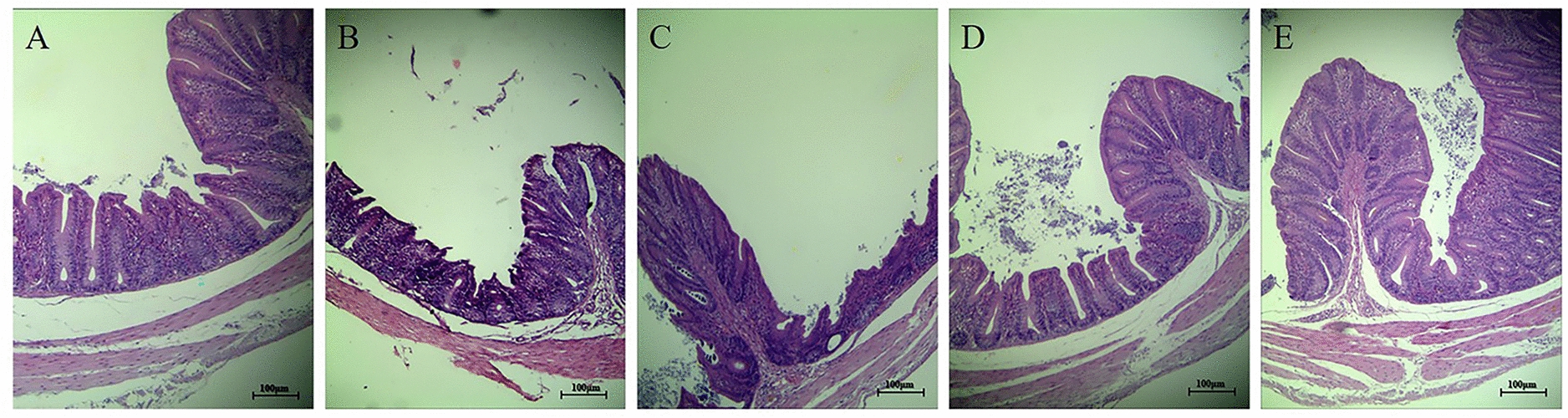
**Histopathological changes in the caeca of chickens from the different groups in Experiment 2 post-challenge with *E. tenella*.** On Day 7 pi, the caeca of chickens from each group in Experiment 2 (*n* = 7) were fixed in neutral buffered formalin (10%), embedded in paraffin, sectioned and stained with haematoxylin and eosin (HE). **A** No histopathological changes were observed in the caecal tissues of the chickens in the PBS group. **B** Histopathological lesions in caecal tissues of the chickens in the infection control group displayed detachment of the intestinal villi, necrosis of the epithelial cells and congestion of the veins. In the **C** L/EA + L/LB(+)and **D** M/EA + M/LB(+)groups, the structures of caecal tissues were relatively complete, and a small number of red blood cells were observed. **E** In the H/EA + H/LB(+)group, no obvious histopathological changes to the caecal tissues were observed, and the intestinal villi were arranged regularly.

No histopathological changes were observed in the caecal tissues in the PBS group, and the intestinal villi were arranged regularly (Figure 13A). Compared with the PBS group, there were clear histopathological lesions in the caecal tissues from the chickens in the *E. tenella-*infected control group (Figure 13B), including detachment of the intestinal villi, necrosis of the epithelial cells and congestion of the veins. In the L/EA + L/LB(+) (Figure 13C) and M/EA + M/LB(+) groups (Figure 13D), the structures of the caecal tissues were relatively complete, and a small number of red blood cells were observed. Importantly, no obvious histopathological changes were found in the caecal tissues of the chickens from the H/EA + H/LB(+) group (Figure 13E), and their intestinal villi were relatively complete and arranged regularly. The above results showed that oral gavage with EA combined with LB(+) provided better anticoccidial effects, and the H/EA + H/LB(+) group displayed the most significant effects against *E. tenella* infection.

## Discussion

Avian coccidiosis is caused by the intestinal protozoan *Eimeria*, which is widely found on chicken farms all over the world. *E. tenella* infection leads to severe intestinal inflammatory injury. In recent years, the relationship between innate immune responses stimulated by *Eimeria* parasites and inflammation have been a hot research topic. ChTLR15 is a unique type of innate immune receptor that recognizes fungal and bacterial secretory proteases [20]. ChTLR15 is unique to poultry; until now, its ligand has not been clear, as the known classical TLR agonists cannot activate it [21]. Our previous study showed that *E. tenella* sporozoites specifically triggered activation of the ChTLR15/NF-κB signalling pathway in vitro [4]. Moreover, the results from ChTLR15 knockdown and overexpression experiments showed that dynamic changes in all of the components in the ChNLRP3/ChIL-1β pathway led to dynamic changes in the ChTLR15/NF-κB pathway [4], which clearly proves that ChTLR15 effectively regulates the expression of key molecules in the ChNLRP3/ChIL-1β pathway.

After learning that the ChTLR15/ChNLRP3 inflammatory signalling pathway is one of the key inflammatory pathways involved in the process of *Eimeria* infection, we were then interested in the signalling pathways that are capable of regulating the ChTLR15/ChNLRP3 pathway and attenuating the intestinal inflammatory injury caused by *Eimeria*. It is widely accepted that the antioxidant signalling pathway Nrf2/HO-1 can inhibit oxidative stress and inflammatory injury by activating the expression of antioxidant response elements (AREs) [22, 23]. Moreover, the Nrf2/HO-1 pathway has been shown to regulate the TLR/NF-κB pathway [24]. Recent studies have reported that inonotus obliquus polysaccharide (IOP) can protect against *Toxoplasma gondii* infection, and this is closely related to activation of the Nrf2/HO-1 pathway and a reduction in TLR-mediated excessive inflammation through ARE activation and proinflammatory factor inhibition [25, 26].

In recent years, a variety of Chinese herbal extracts, such as *Shi Ying Zi* [27], *berberine* [28], the mushrooms *Agaricus subrufescens* and *Pleurotus ostreatus* [29], *Cinnamomum verum* [30] and *Canary rue* [31], have been reported to show anticoccidial effects to some extent. EA, a polyphenol extracted from certain medicinal plants, is a member of the tannin family that possesses several biological properties, including antioxidant and anti-inflammatory properties [32, 33]. However, the abilities of EA to relieve inflammatory intestinal injury caused by *Eimeria* remain elusive. In addition, lactic acid bacteria have been reported to regulate antioxidant stress [34]. Therefore, in the present study, *L. brevis* 23017, which was previously shown to ameliorate intestinal inflammation and alleviate oxidative stress [9], and EA were selected as activators of the Nrf2/HO-1 antioxidant pathway to explore the effects of *L. brevis* 23017 alone or in combination with EA on the cross-regulation of the Nrf2/HO-1 and ChTLR15/ChNF-κB-ChNLRP3/ChIL-1β pathways in vivo. The results showed that live *L. brevis* 23017, especially at a high dosage, effectively activated the Nrf2/HO-1 signalling pathway.

To explore whether LB(+) combined with EA displayed a greater capability to trigger activation of the Nrf2/HO-1 signalling pathway, animal Experiment 2 was carried out, and three dosages of EA were administered. Compared with the M/EA + M/LB(+) and L/EA + L/LB(+) groups, the mRNA levels of the related molecules in the Nrf2/HO-1 signalling pathway and the protein levels of Nrf2 and the antioxidant enzymes in the caecal tissues of chickens in the H/EA + H/LB(+) group showed the strongest antioxidant effects (*P* < 0.01), suggesting that the antioxidant capacity activated by oral administration of LB(+) was significantly enhanced after combination treatment with EA.

To investigate whether activation of the Nrf2 signalling pathway inhibited intestinal inflammatory injury caused by *E. tenella* by regulating the ChTLR15/ChNLRP3 pathway, chickens in Experiments 1 and 2 were infected with *E. tenella* by oral gavage with LB(+), LB(−) alone and LB(+) in combination with EA. The mRNA expression levels of key molecules in the ChTLR15/ChNLRP3 pathway, the protein levels of ChTLR15, ChNLRP3 and ChIL-1β, and the protein levels of the oxidant enzyme MDA in the three LB(+) groups were significantly downregulated compared with those in the *E. tenella* infection control group. These results were reflected by the observed anticoccidial effects, including decreased lesion scores, alleviated histopathological changes in the caeca, increased body weight gain and decreased oocyst ratios. Importantly, the anti-inflammatory and anticoccidial effects on the chickens in the H/LB(+) + H/EA group were the best. In addition, in vitro experiments showed that the selected dosages of EA in animal Experiment 2 did not display direct roles on the development of *E. tenella* sporozoites (data not shown). The above results indicated that oral gavage of LB(+) combined with EA effectively enhanced the activation of the Nrf2/HO-1 pathway, effectively inhibited the ChTLR15/ChNLRP3 inflammatory pathway triggered by *E. tenella*, and thus provided anti-inflammatory effects. The results from the present study are consistent with those of other reports, which showed that the combination of probiotics and herbs had strong potential functions [35, 36]. A possible explanation for the present results may be related to the following questions. Was the intestinal microbiota changed after the combination treatment of EA and *L. brevis* 23017? And was a novel EA-derived or *L. brevis* 23017-derived material produced? Recently, two reports showed that feed enzymes had the potential to reverse unfavourable caecal fermentation patterns [37] and ameliorate the deleterious effects of coccidiosis on intestinal health [38]. Thus, does the combination of EA and *L. brevis* 23017 influence intestinal enzymes? All of the above questions will be further analysed in our subsequent research work.

Taken together, these results showed that oral gavage with a high dosage of live *L. brevis* 23017 and ellagic acid produced significant anti-inflammatory effects by regulating the ChTLR15/NLRP3/IL-1β pathway in chickens. Therefore, the combination of this traditional Chinese medicine and lactic acid bacteria with excellent performance is a promising way to prevent and control *Eimeria* and restrict the use of anticoccidial drugs.
